# The *Arabidopsis thaliana* elongator complex subunit 2 epigenetically affects root development

**DOI:** 10.1093/jxb/erv230

**Published:** 2015-05-21

**Authors:** Yuebin Jia, Huiyu Tian, Hongjiang Li, Qianqian Yu, Lei Wang, Jiri Friml, Zhaojun Ding

**Affiliations:** ^1^The Key Laboratory of Plant Cell Engineering and Germplasm Innovation, Ministry of Education, College of Life Science, Shandong University, Jinan 250100, China; ^2^Institute of Science and Technology Austria, 3400 Klosterneuburg, Austria; ^3^Key Laboratory of Plant Molecular Physiology, Institute of Botany, Chinese Academy of Sciences, Beijing 100093, China

**Keywords:** Auxin ELP2, epigenetics, root.

## Abstract

The elongator complex subunit 2 (ELP2) protein, one subunit of an evolutionarily conserved histone acetyltransferase complex, has been shown to participate in leaf patterning, plant immune and abiotic stress responses in *Arabidopsis thaliana*. Here, its role in root development was explored. Compared to the wild type, the *elp2* mutant exhibited an accelerated differentiation of its root stem cells and cell division was more active in its quiescent centre (QC). The key transcription factors responsible for maintaining root stem cell and QC identity, such as AP2 transcription factors *PLT1* (*PLETHORA1*) and *PLT2* (*PLETHORA2*), GRAS transcription factors such as *SCR* (*SCARECROW*) and *SHR* (*SHORT ROOT*) and WUSCHEL-RELATED HOMEOBOX5 transcription factor *WOX5*, were all strongly down-regulated in the mutant. On the other hand, expression of the G2/M transition activator *CYCB1* was substantially induced in *elp2*. The auxin efflux transporters PIN1 and PIN2 showed decreased protein levels and PIN1 also displayed mild polarity alterations in *elp2*, which resulted in a reduced auxin content in the root tip. Either the acetylation or methylation level of each of these genes differed between the mutant and the wild type, suggesting that the ELP2 regulation of root development involves the epigenetic modification of a range of transcription factors and other developmental regulators.

## Highlight

The *elp2* mutant affected the expression of key transcription factors and *CYCB1* through either acetylation or methylation, and also altered auxin polar transport and reduced auxin content in root.

## Introduction

The maintenance of a functional root system makes an important contribution to a plant’s capacity to adapt to instability in its growing environment. At the root apical meristem, root stem cells deliver the cells that differentiate to form the various tissue types found in the root (Dolan *et al.*, 1993). Stem cell identity within the root meristem is maintained by signals generated from cells in the quiescent centre (QC) (Vernoux and Benfey, 2005). The mitotically less active QC, together with its surrounding stem cells, constitutes the ‘root stem cell niche’, a structure that is essential for the maintenance of root growth (Sabatini *et al.*, 2003). The specification of cell identity within the root stem cell niche is determined by the activity of a number of transcription factors: these include members of both the AP2 (*PLT*s) and GRAS (*SCR* and *SHR*) transcription factor families (Helariutta *et al.*, 2000; Sabatini *et al.*, 2003; Aida *et al.*, 2004; Galinha *et al.*, 2007). PLT proteins are not only involved in QC specification during embryogenesis but also have a more general role in the maintenance of root stem cell niche identity. In loss-of-function mutants, the embryonic root does not form, while the expression of *PLT* in the shoot is sufficient to specify root identity in shoot (Aida *et al.*, 2004; Galinha *et al.*, 2007). Both SHR and SCR affect radial patterning in the root, and are also required for QC specification and root stem cell niche maintenance (Helariutta *et al.*, 2000; Sabatini *et al.*, 2003). The homeodomain transcription factor *WOX5* is specifically expressed in the QC in the wild-type (WT) plant. Its loss-of-function results in the formation of enlarged QC cells and promoted differentiation of the distal stem cells, but has no observable effect on either root growth or the size of the more proximal meristem cells; its over-expression blocks the differentiation of distal stem cells, thereby inducing the formation of multiple layers of distal stem cells. The primary function of *WOX5* appears to be to maintain distal stem cell identity (Sarkar *et al.*, 2007).

Root stem cell niche identity is also regulated by auxin and cytokinin (Dello Ioio *et al.*, 2007; Ding and Friml, 2010). Auxin initiates, organizes and maintains the root stem cell niche (Benjamins and Scheres, 2008), while the effect of cytokinin is to modulate the auxin pathway or polar auxin transport (Dello Ioio *et al.*, 2008; Ruzicka *et al.*, 2009). Auxin and cytokinin act antagonistically to define root apical meristem size by promoting, respectively, cell division and differentiation (Dello Ioio *et al.*, 2007). The maintenance of the root stem cell niche depends on the establishment of an auxin concentration gradient, which peaks at the stem cell niche. The stability of this gradient relies on both PIN-mediated distribution and its local synthesis (Blilou *et al.*, 2005; Grieneisen *et al.*, 2007; Tian et al., 2013, 2014).

The eukaryotic elongator complex consists of an ELP1, ELP2 and ELP3 core and an accessory ELP4, ELP5 and ELP6 subcomplex (Wittschieben *et al.*, 1999; Kim *et al.*, 2002; Nelissen *et al.*, 2010; Versees *et al.*, 2010). It participates in acetylation, methylation/demethylation, exocytosis and tRNA modification (Kim *et al.*, 2002; Creppe *et al.*, 2009; Chen *et al.*, 2011; Defraia *et al.*, 2013; Wang *et al.*, 2013). In *Arabidopsis thaliana*, it mediates part of the response to biotic and abiotic stress, as well as being involved in the determination of leaf patterning (Nelissen *et al.*, 2005, 2010; Zhou *et al.*, 2009; Xu *et al.*, 2011; Defraia *et al.*, 2013). Previous studies also showed that the *elp1, elp3, elp4* mutants have defected root growth, however, the detailed mechanism was not well studied (Nelissen *et al.*, 2005). Here, we mapped *elp*2 mutant according to its root stem cell defective phenotype and thoroughly investigated the role of *ELP2* on root development.

## Materials and methods

### Plant materials and growth conditions

The transgenic *A. thaliana* lines used were *WOX5:GFP* (Sarkar *et al.*, 2007); *CYCB1;1:GUS* (Colon-Carmona *et al.*, 1999); *DR5:GUS* (Ulmasov *et al.*, 1997); *DR5rev:GFP* (Benkova *et al.*, 2003); *PIN1:PIN1-GFP* (Benkova *et al.*, 2003); *PIN2:PIN2-GFP* (Blilou *et al.*, 2005); *PLT1*_***pro***_ *:CFP*, *PLT1*_***pro***_ *:PLT1-YFP*, *PLT2*_***pro***_ *:CFP*, and *PLT2*_***pro***_ *:PLT2-YFP* (Kornet and Scheres, 2009); QC25 (Sabatini *et al.*, 2003); *SCR*_***pro***_ *:SCR-GFP* (Di Laurenzio *et al.*, 1996); *SHR*_***pro***_ *:SHR-GFP* (Nakajima *et al.*, 2001).The mutant *elp2*, and *elp6* are from an ethyl methanesulfonate mutagenized populations, *elp1 (SALK_004690*) and *elp4 (SALK_079193*) are from the Arabidopsis Stock Center, which are all in the Columbia (Col-0) background (Nelissen *et al.*, 2005; Zhou *et al.*, 2009;). Seeds were surface-sterilized by exposure to chlorine gas, sown on solidified Murashige and Skoog (1962) medium (MS medium), held for 2 d at 4ºC, then raised under a 16h photoperiod at 19ºC for a further 5 d.

### TAIL-PCR

DNA was extracted from leaves of WT ecotype Col-0 and the *elp2* mutant using a CTAB-based method. The primary PCR was primed with the T-DNA specific *LBa1* and a degenerate *AD* primer (sequences given in Supplementary Table S1). The secondary PCR was based on *LBb1.3* and an *AD* primer, using a diluted aliquot of the primary PCR as the template. The tertiary PCR was based on *LBb1* and an *AD* primer, using a diluted aliquot of the secondary PCR as the template. The amplicons were separated by agarose gel electrophoresis and the *dsr1* specific band compared to the WT (Col) was sequenced to find the T-DNA boundary sequence.

### GUS, EdU and lugol staining

Staining of seedling roots for β-glucuronidase (GUS) activity was carried out by incubation at 37°C in 0.05M NaPO_4_ buffer (pH 7.0), 5mM K_3_Fe(CN)_6_, 5mM K_4_Fe(CN)_6_ and 2mM X-glucuronide. Once the colour had developed, the material was passed through an ethanol series (70%, 50% and 20%) before mounting in 70% chloral hydrate plus 10% glycerol. EdU staining was performed following the protocol supplied with the Click-iT® EdU Alexa Fluor® 555 Imaging kit (Invitrogen). Detection of starch granules in the root tip followed staining in Lugol’s solution for 1–2min, after which the material was mounted in chloral hydrate as above.

### RNA analysis

Seedlings were grown on MS medium for 5 d, after which the distal 2mm of the roots were harvested for RNA extraction. Total RNA was isolated using a RNeasy® Mini Kit (Qiagen) and the first strand of cDNA was synthesized from a 2 µg aliquot using a Transcriptor First Strand cDNA Synthesis kit (Roche) following the manufacturer’s protocol. Quantitative real-time PCRs (qRT-PCRs) were based on the CFX Connect^TM^ Real-Time System (Bio-Rad) and FastStart Universal SYBR Green Master mix (Roche). Three biological replicates were included, each of which was represented by three technical replicates. *AtACTIN2* was used as the reference sequence.

### Chromatin immunoprecipitation (ChIP) assay

ChIP was used to quantify the acetylation level of the set of selected genes. The assay was based on an ~3g sample of two-week-old seedlings, following the Wang *et al.*, (2013) protocol. After *in vivo* cross-linking and tissue lysis, DNA was sheared by sonication and evaluated by agarose gel electrophoresis. The extracted nucleosomes were precleared using 40 µl protein A agarose beads (Invitrogen), after which was added 2–3 µg Ac-Histone H3 (Lys-9/14) antibody (sc-8655-R; Santa Cruz Biotechnology) before incubation at 4ºC overnight. After de-cross-linking, DNA was extracted and precipitated using triple volumes of ethanol, NaAc, and glycogen. The resulting DNA was used as the template for a qRT-PCR, based on primers detailed in Supplementary Table S1. The acetylation level in each sample was normalized to both input DNA and *AtACTIN2* as described elsewhere (Mosher *et al.*, 2006). The Chromatin immunoprecipitation (ChIP) assay was done three times independently. The data represent mean values with their associated SD (n=3), *P* <0.05.

### Bisulfite sequencing

Genomic DNA was extracted with a CTAB protocol from ~1g seedlings harvested from plants raised for two weeks on MS medium. About 2 µg DNA was treated with an EpiTect® Bisulfite kit (Qiagen). Strand-specific and bisulfite-specific primers (see Supplementary Table S1) were designed using MethPrimer software (http://www.urogene.org/methprimer/). The amplicons were introduced into a pEASY-T1 simple vector (Tiangen) for sequencing, and the resulting sequences were analysed using DNAMAN software. Each sample was represented by three biological replicates.

### Immunolocalization assay

PIN1 and PIN2 were immunolocalized as described elsewhere (Dai *et al.*, 2012).

### Phenotypic analysis, microscopy, statistics

Seedlings grown on MS were scanned using EPSON PERFECTION V700 PHOTO, and root length was measured by Image J. Root meristems were analysed on seedlings mounted in HCG solution (Chloral hydrate:water:glycerol=8:3:1). Root meristem size was assessed as the cell number from the QC to the first elongating cell in the cortex cell file. Root micrographs were photographed using OLYMPUS BX53. Confocal imaging was obtained using an LSM-700 laser-scanning confocal microscope (Zeiss). Five-day-old seedling root tips were stained in propidium iodide.

Data presented are mean values of at least three biological repeats with SD. The statistical significance was analysed by Student’s t-test analysis.

## Results

### The *elp2* mutant shows defective root stem cell niche maintenance

**Fig. 1.**
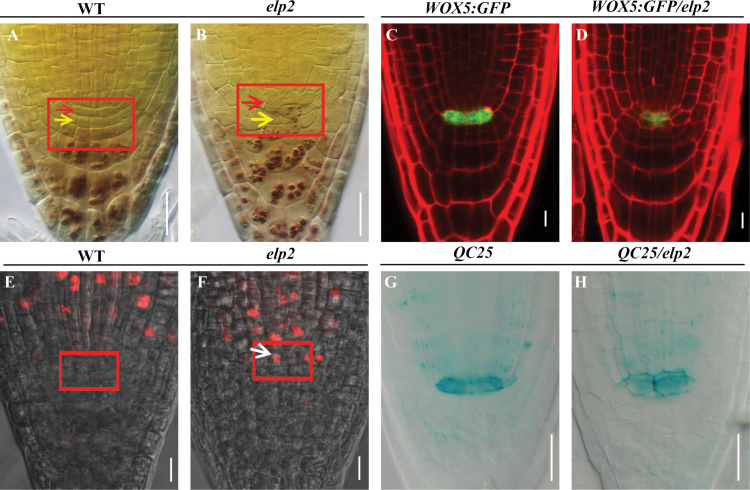
The *elp2* mutant is defective with respect to root stem cell niche maintenance. Lugol stained (A) WT and (B) *elp2* roots. Cell division in the mutant QC is enhanced (red arrowheads), while its distal stem cells are deficient in starch (yellow arrowheads). The root stem cell niche is shown boxed. Confocal micrographs of (C) WT and (D) *elp2* plants expressing the transgene *WOX5:GFP*; the level of expression in the mutant is lower than in WT. Confocal micrographs illustrating the incorporation of EdU into (E) WT and (F) *elp2* QC cells. The fluorescent signals show that the mutant QC is in a state of active division (white arrowheads). The QC is shown boxed. *QC25* is expressed at a higher level in (G) the WT than in (H) the *elp2* mutant. Bars: 20 µm (A, B, G, H); 10 µm (C–F). (This figure is available in colour at JXB online.)

A search for T-DNA mutants displaying a defective root stem cell niche maintenance phenotype identified *drs1*, a mutant that exhibited enhanced root distal stem cell differentiation and increased mitotic activity in its QC (Fig. 1). When five-day-old seedlings were exposed for 24h to EdU [a thymidine analogue used to mark S-phase progression (Vanstraelen *et al.*, 2009)], a stronger level of fluorescence was observed in the nuclei of mitotically active cells in the mutant than in WT, due to the coupling of EdU with Aexa Fluor 555. This assay indicated that mitotic activity was enhanced in *elp2* QC cells (Fig. 1E, F). The QC specific transcription factor *WOX5* was also strongly down-regulated (Fig. 1C, D), as was the signal produced by the QC marker *QC25* (Fig. 1G, H).

A TAIL-PCR analysis identified that in the mutant, the *ELP2* locus (At1g49540) had been interrupted by the insertion of a T-DNA element within the second intron (Supplementary Figs S1, S2). The hybrid between *drs1* and *elp2* [the latter differs from WT by a G to A transition at the acceptor splice site of the fifth intron (Zhou *et al.*, 2009)], just like the parental mutant lines, displayed both enhanced root distal stem cell differentiation and increased QC division, confirming that the defective root phenotype expressed by *drs1* was caused by a lesion in *ELP2.* We renamed the *elp2* mutant from EMS populations (Zhou *et al.*, 2009) as the *elp2-6*, and renamed the *drs1* as the *elp2-7* following the *elp2-2*, *elp2-3*, *elp2-4*, *elp2-5* indentified previously (Nelissen *et al.*, 2005; DeFraia *et al.*, 2010). The *elp2-7* was used for most of the analysis and termed *elp2* afterwards in this paper. Mutations affecting other subunits of the elongation complex exhibited similar defective root stem cell niche maintenance (Supplementary Fig. S3).

### The *elp2* mutant’s root meristem is reduced in size

**Fig. 2.**
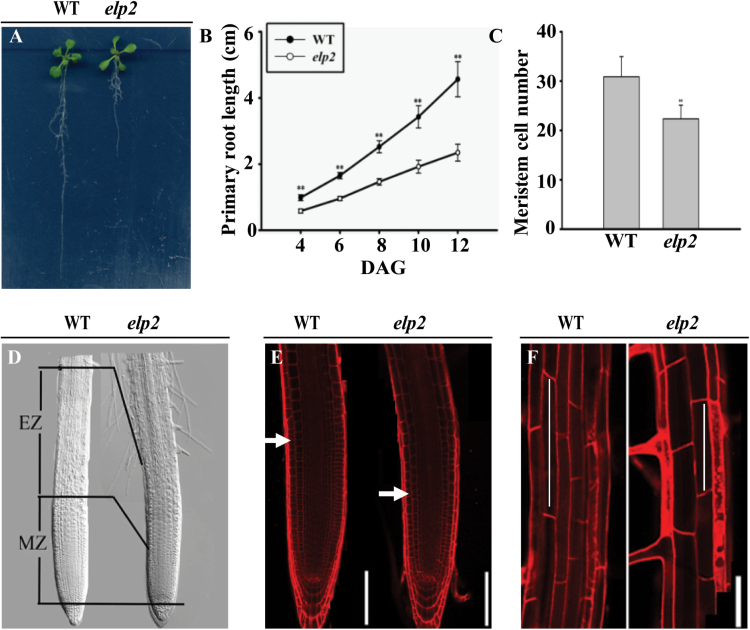
The foreshortening of the roots produced by the *elp2* mutant reflects a reduced level of cell proliferation and cell elongation. (A) Roots of 12-day-old seedlings of WT and *elp2*. (B) Primary root growth of WT and *elp2* seedlings 4 d post germination. The data represent the mean and SD (*n*=40) derived from at least three independent experiments. (C) Variation in root meristem cell number of five-day-old WT and *elp2* seedlings. Cell numbers counted from the QC to the TZ. The data represent the mean and SD (*n*=20) derived from at least three independent experiments. (D) Representative five-day-old WT and *elp2* seedlings illustrating that the latter develop a reduced MZ and EZ. (E) Five-day-old WT and *elp2* seedlings. The cortex TZ is indicated by white arrowheads. (F) Epidermal cell length of the DZ in six-day-old WT and *elp2* seedlings. Bars, 50µm (E, F). **, *P*<0.001 (This figure is available in colour at JXB online.)

In addition to the root stem cell niche identity defect, the *elp2* mutant also produced shorter roots than the WT seedlings (Fig. 2A, B, Supplementary Fig. S4). This shortening was a result of smaller elongation (EZ) and meristem (MZ) zones (Fig. 2D, E). Cell number in the MZ and cell length in the EZ were both reduced compared to the WT (Fig. 2C, F), suggesting that *ELP2* affects both cell proliferation and cell elongation.

### The down-regulation of transcription factors in the *elp2* mutant

**Fig. 3.**
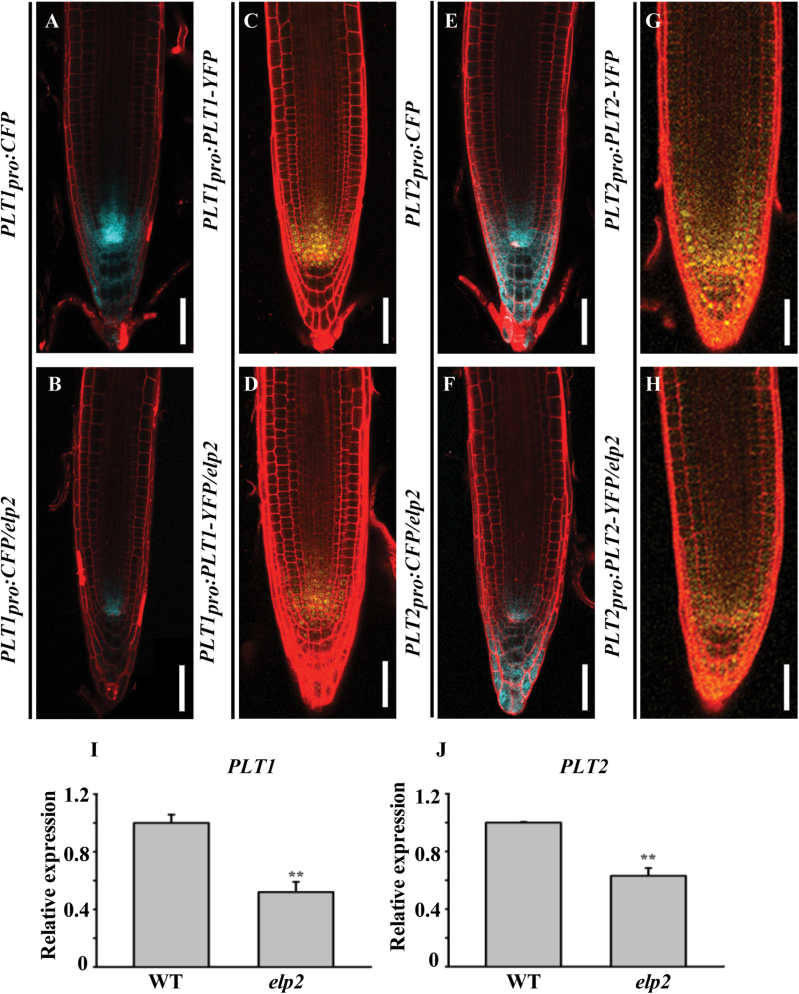
*PLT1* and *PLT2* expression is reduced in the *elp2* mutant. Expression of the transgenes (A, B) *PLT1*_***pro***_ *:CFP*, (C, D) *PLT1*_***pro***_ *:PLT1-YFP*, (E, F) *PLT2*_***pro***_ *:CFP*, (G, H) *PLT2*_***pro***_ *:PLT2-YFP*. qRT-PCR assay of the transcription of (I) *PLT1* and (J) *PLT2*. The data represent mean values with their associated SD (*n=*3); **, *P*<0.001. Bars, 50 µm (A–H). (This figure is available in colour at JXB online.)

**Fig. 4.**
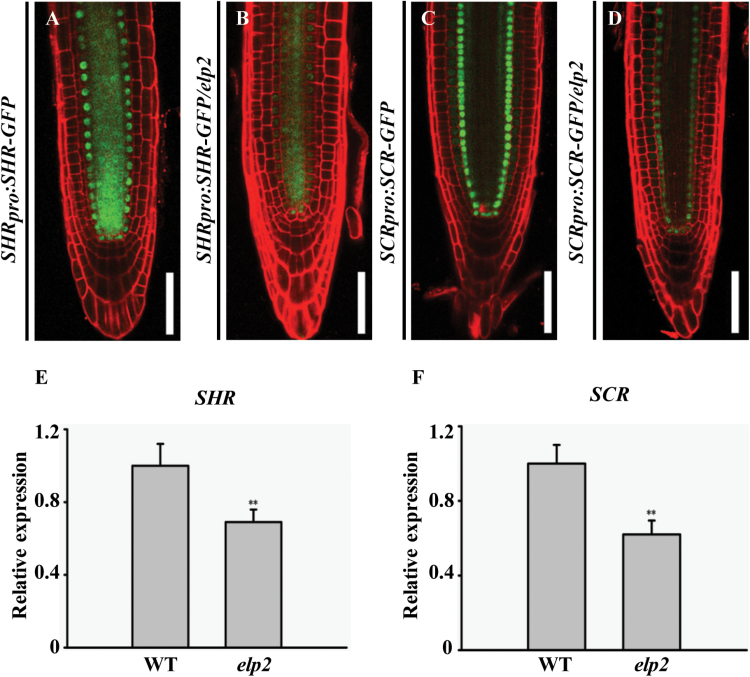
*SHR* and *SCR* expression is reduced in the *elp2* mutant. Expression of the transgenes (A, B) *SHR*_***pro***_ *:SHR-GFP*, (C, D) *SCR*_***pro***_ *:SCR-GFP*. qRT-PCR assay of the transcription of (E) *SHR* and (F) *SCR*. The data represent mean values with their associated SD (*n=*3); **, *P*<0.001. Bars, 50 µm (A–D). (This figure is available in colour at JXB online.)

To determine whether the effects of ELP2 on root stem cell niche maintenance is dependent on the well characterized root stem cell niche-defining transcription factors, we examined the expression of AP2 transcription factors such as *PLT1* and *PLT2* and GRAS transcription factors such as *SCR* and *SHR* (Helariutta *et al.*, 2000; Sabatini *et al.*, 2003; Aida *et al.*, 2004; Galinha *et al.*, 2007) in *elp2*. The expression of both *PLT1*_***pro***_ *:PLT1-YFP* (Fig. 3C, D) and *PLT2*_***pro***_:*PLT2-YFP* (Fig. 3G, H) was strongly reduced in *elp2* compared to the levels achieved in the WT, and the *PLT1*_***pro***_ *:CFP* and *PLT2*_***pro***_:*CFP* behaved similarly (Fig. 3A, B, E, F). Consistently, a qRT-PCR assay showed that the expression level of *PLT1* and *PLT2* was lower in the *elp2* mutant than in the WT (Fig. 3I, J). Thus the evidence was that ELP2 affected the transcription of both *PLT1* and *PLT2*. The expression of *SHR* and *SCR* was investigated by comparing the expression of both *SCR*_***pro***_ *:SCR-GFP* and *SHR*_***pro***_ *:SHR-GFP* in both *elp2* and WT. Both transgenes were strongly down-regulated in the mutant (Fig. 4A–D), a result that was confirmed by a qRT-PCR assay (Fig. 4E, F).

### *ELP2* is required for cell-cycle progression in the root

**Fig. 5.**
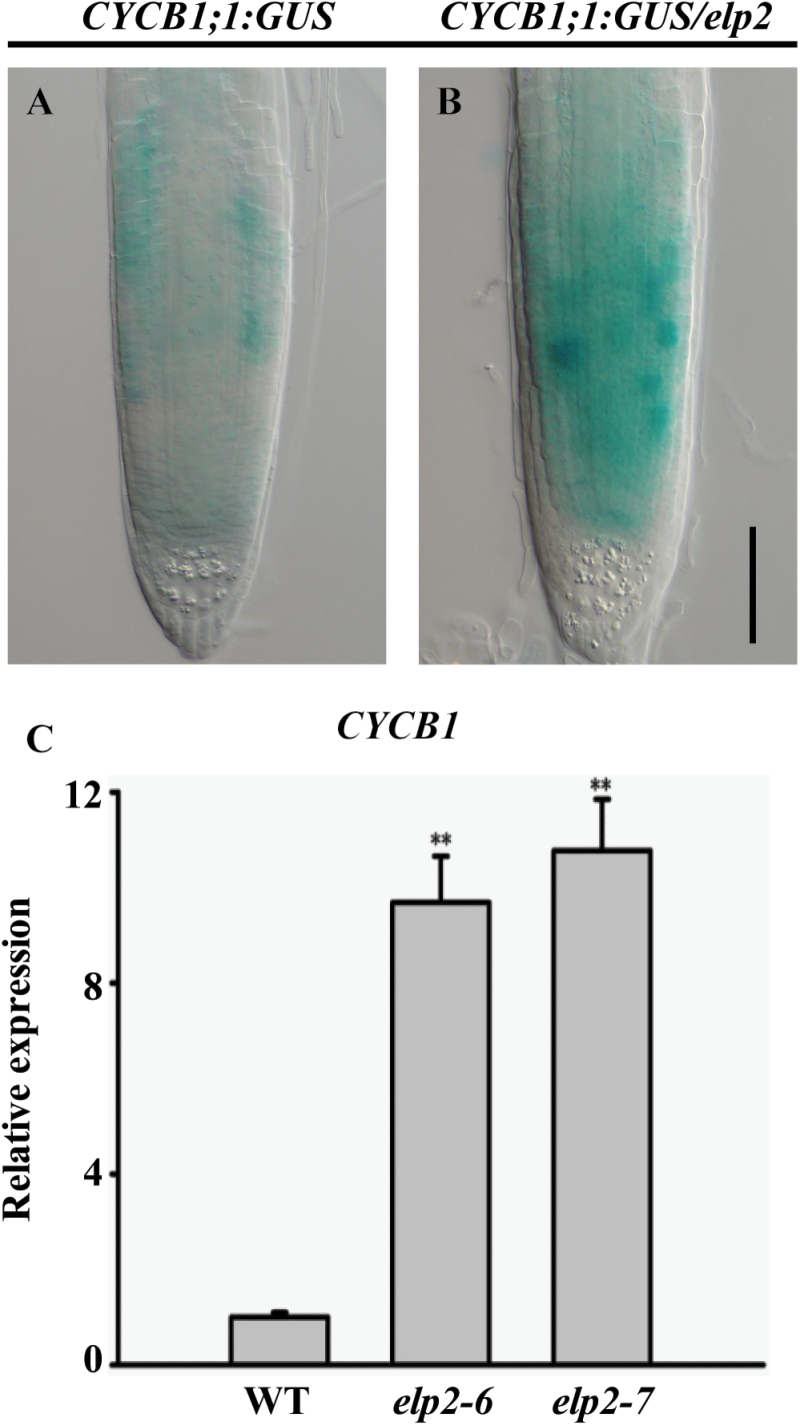
*CYCB1* expression is increased in the *elp2* mutant. (A, B) Expression of the *CYCB1;1:GUS* transgene, (C) transcription of *CYCB1* assayed by qRT-PCR. The data represent mean values with their associated SD, (*n=*3); **, *P*<0.001. Bars, 50 µm (A, B). (This figure is available in colour at JXB online.)

The increased QC division and reduced root meristem cell numbers in *elp2* suggest that ELP2 is required for proper cell-cycle progression in the root. Cells at the G2-M phase were visualized by the transgene *CYCB1;1:GUS* which reflects the cell cycle activity, however timely degradation of proteins is necesssary to ensure the access of M phase (Colon-Carmona *et al.*, 1999). In tobacco, the nondegradable Cyclin B1 results in abnormal cytokinesis and endomitosis (Weingartner *et al.*, 2004). We observed that accumulation of the CYCB1;1:GUS signal in root meristem of *elp2* was much stronger than that in the WT (Fig. 5A, B). In addition, stronger staining in the root QC of *elp2* is consistent with the enhanced cell division in QC (Fig. 5A, B). Next, using qRT-PCR assays, we compared the *CYCB1;1* expression level in the *elp2* mutation and WT. In line with GUS staining, the *elp2* mutant has high *CYCB1;1* expression in the root (Fig. 5C). Therefore, our results showed that *ELP2* is required for cell-cycle progression in the root.

### The *elp2* mutant has a reduced auxin content in root tip

**Fig. 6.**
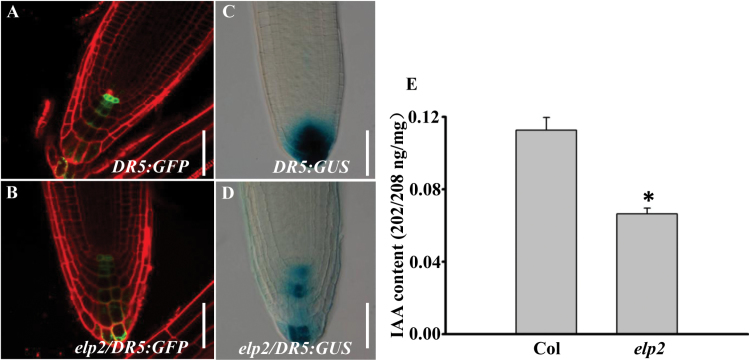
The *elp2* mutant exhibits reduced auxin contents in roots. (A, B) The mutant shows a reduced level of *DR5rev:GFP* expression in the root than the WT. (C, D) The mutant shows a reduced level of *DR5:GUS* expression in the root than the WT. (E) IAA content measurement of roots on 14-day-old seedlings of col and *elp2.* The data represent mean values of three independent biological repeats with their associated SD (*n=*3) *, *P*<0.05. Bars, 50 µm (A–D). (This figure is available in colour at JXB online.)

Auxin gradients (Vanneste and Friml, 2009) are central to the robust development of the primary root in *Arabidopsis*, and the auxin gradient in the root tip could be instructive for the patterning of root distal stem cell (DSC) niches (Tian et al., 2013, 2014). To substantiate if the root defective phenotypes in *elp2* are resulting from the changed auxin signalling in root tips, *DR5rev:GFP* and *DR5:GUS* synthetic auxin response reporters (Friml *et al.*, 2003) were used to assay auxin signalling. The signal from both reporters was markedly lower in the mutant than in the WT root tip (Fig. 6A–D). The GUS enzyme activity assay using the MUG as the substrate further confirmed this difference (data not shown). Furthermore, we measured the free IAA content in roots of the *elp2* mutant. Consistently, the *elp2* mutant has reduced IAA content in roots compared to the wild-type control (Fig. 6E).

### Auxin polar transport was affected in *elp2*

**Fig. 7.**
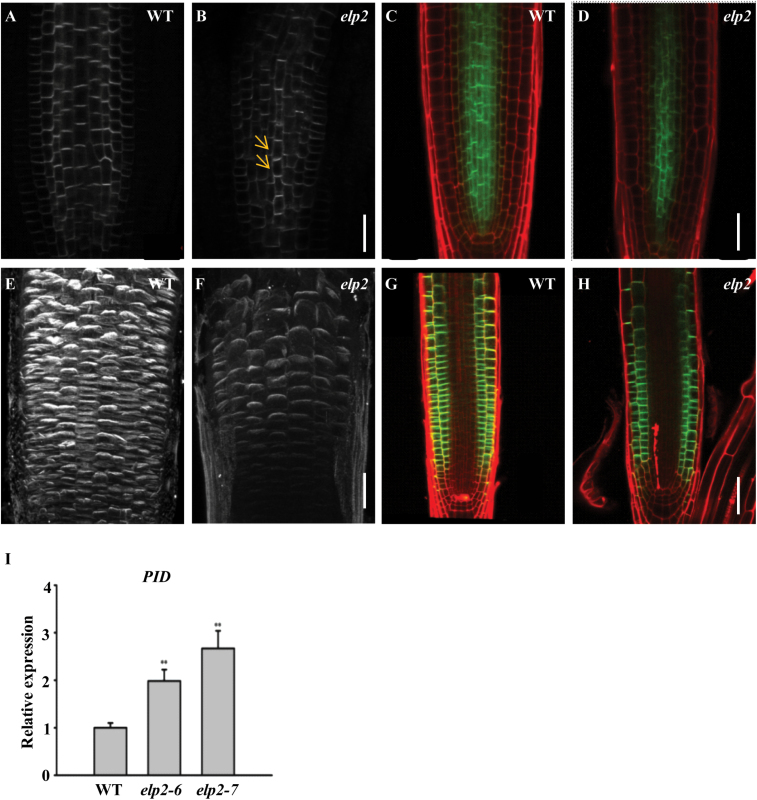
PIN1 and PIN2 localization and expression in the *elp2* mutant. (A, B) PIN1 immunolocalization in five-day-old roots. (C, D) Expression and polarity of PIN1:PIN1-GFP in the WT and mutant root. (E, F) PIN2 immunolocalization in five-day old roots. (G, H) Expression and polarity of PIN2:PIN2-GFP in the WT and mutant root. (I) *PID* transcription assayed by qRT-PCR. The data represent mean values with their associated SD (*n=*3); **, *P*<0.001. Bars, 50 µm (A–H). (This figure is available in colour at JXB online.)

To address if the reduced auxin signalling in root tips of *elp2* was a result of the affected polar auxin transport, we examined the polar localization of auxin efflux carriers such as PIN1 and PIN2 (Petrasek *et al.*, 2006), which mediate auxin distribution in plants, including to the roots (Blilou *et al.*, 2005; Wisniewska *et al.*, 2006). Basal localized PIN1 in the stele and basal localized PIN2 in the cortex contribute to auxin transportation from shoot to root, thus contributing to maximum auxin formation in root tips and regulating root growth (Galweiler *et al.*, 1998; Friml *et al.*, 2003; Blilou *et al.*, 2005; Wisniewska *et al.*, 2006). However, in *elp2*, basal localization of PIN1 is less polar, and the increased lateral localization of PIN1 was observed from both our immunolocalization examinations with the anti-PIN1 antibody and confocal examinations with the *PIN1-GFP* line (Fig. 7A–D). Though apical PIN2 in the epidermis displays WT polarity in *elp2* (Fig. 7E–H), both the protein expression levels of *PIN1* and *PIN2* were reduced in *elp2* (Fig. 7C, D, G, H).

Previous studies have already demonstrated that PP2A and PINOID partially colocalize with PINs and act antagonistically on the phosphorylation state of PINs hydrophilic loop (Benjamins *et al.*, 2001; Michniewicz *et al.*, 2007; Huang *et al.*, 2010). *35S:PID* seedlings have basal to apical localization of PIN1 (Michniewicz *et al.*, 2007). Thus, we examined the *PINOID* expression in the *elp2* mutant. We found the *elp2* mutant to have a consistently higher expression level of *PINOID* compared with the WT control(Fig. 7I).

### ELP2 affects the histone acetylation levels of several key transcription factor genes

**Fig. 8.**
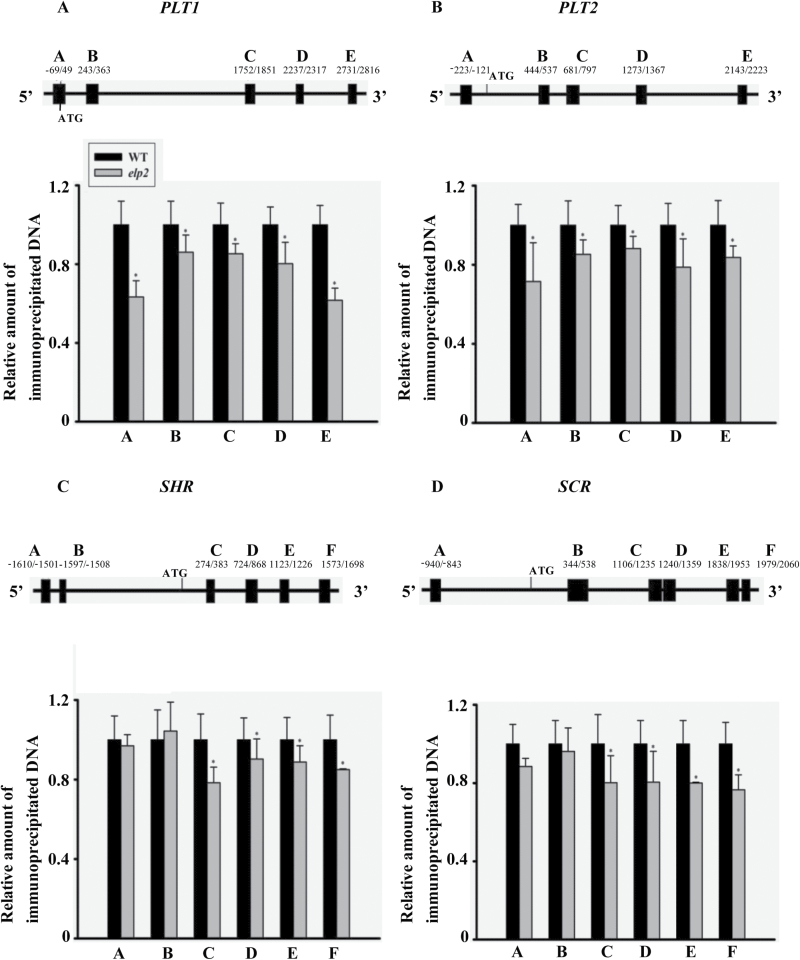
Histone H3 acetylation levels in (A) *PLT1*, (B) *PLT2*, (C) *SHR* and (D) *SCR*. The placement of the primers is indicated. The relative amount of immunoprecipitated chromatin fragments in the *elp2* mutant, as determined by qRT-PCR, was compared to that produced in the WT. The data represent mean values with their associated SD (*n=*3); *, *P*<0.05.

The histone acetyltransferase of ELP3 has been confirmed both in yeast and plants (Winkler *et al.*, 2002; Nelissen *et al.*, 2010). To address whether the reduced expression level of several key transcription factors encoding genes such as *PLT1*, *PLT2*, *SCR* and *SHR* in *elp2* may be associated with the reduced acetylation levels, we performed CHIP-PCR using an antibody specific for histone H3 acetylated at Lys-9 and -14 (H3K9/14ac). CHIP-PCR was performed to verify whether the reduced expression of *PLT1*, *PLT2*, *SCR* and *SHR* in the mutant was correlated with the altered acetylation level. The assay based on primers targeting the coding region and the 5ʹ-UTR of *PLT1* and *PLT2* showed that acetylation in this part of the gene was markedly reduced in the mutant (Fig. 8), while the acetylation level of the internal reference gene *ACTIN2* was not changed (Supplementary Fig. S5A). A similar result was obtained with respect to the *SHR* and *SCR* coding regions which show reduced acetylation, but there was no difference between the WT and the mutant within promoter regions of both genes (Fig. 8). In addition, we also found a reduced acetylation level in 3ʹ coding region of *PIN1*, consistent with the reduced expression level of *PIN1* (Supplementary Fig. S5B).

### ELP2 is required for the DNA methylation of *CYCB1* but not of *PID*, *SHR* or *SCR*

**Fig. 9.**
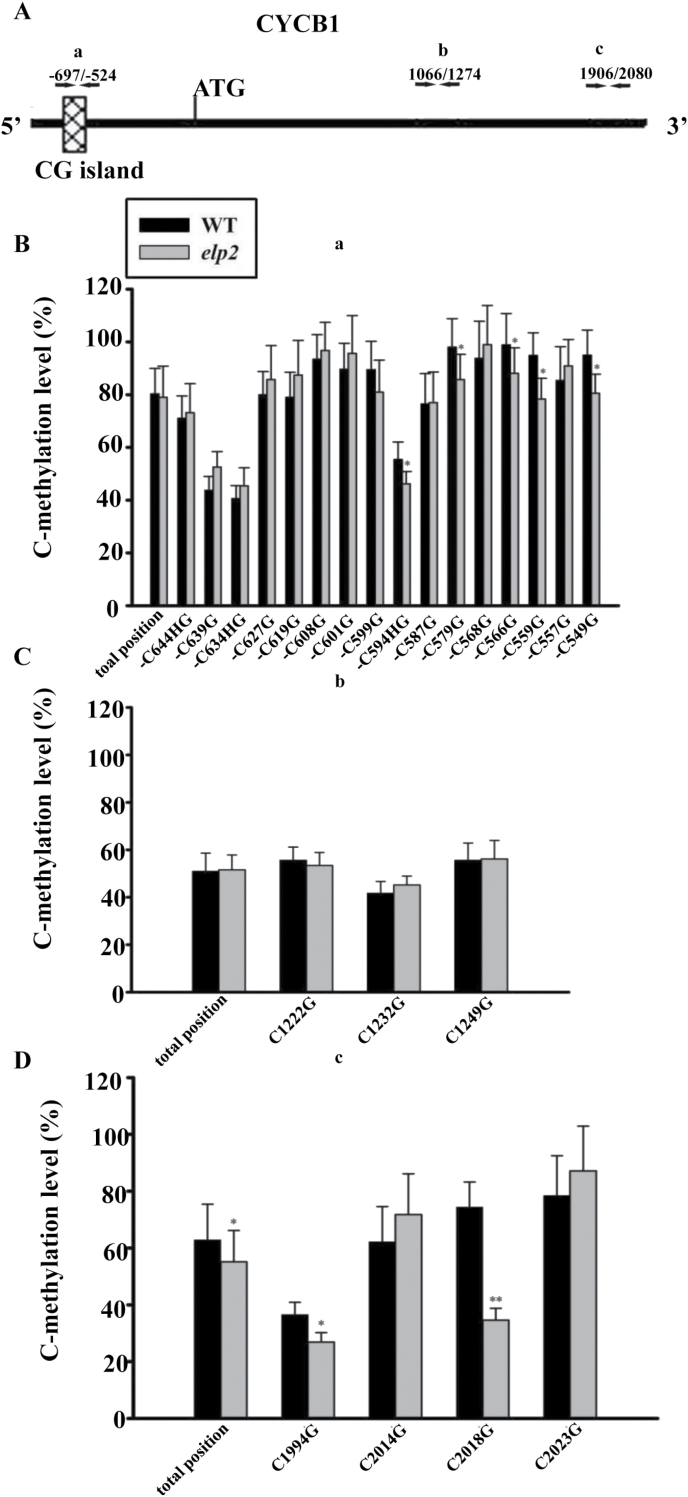
DNA methylation level is altered in the *elp2* mutant. (A) Placement of the primers is indicated. The rectangular box represents a CG island. (B–D) The methylation level of amplified fragments a (B), b (C) and c (D). The DNA was extracted from three biological replicates of both WT and *elp2*. Three replicates of 60 clones derived from each of WT and *elp2* were bisulfite-sequenced to assess their methylation level. The data represent mean values with their associated SD (*n=*3); *, *P*<0.05, **, *P*<0.001.

ELP2 has recently been reported to be involved in somatic DNA demethylation/methylation, thus regulating pathogen-induced transcriptome reprogramming and plant immune responses (Wang *et al.*, 2013). To address the reduced expression levels of transcription factors such as *SCR* and *SHR*, the increased expression of *PID*, and whether the increased expression of the cell cycle gene *CYCB1* in *elp2* are associated with the altered methylation levels, DNA methylation levels in *CYCB1*, *PID*, *SHR* and *SCR* were estimated by bisulfite sequencing. The level of methylation throughout both the promoter and coding regions of *PID*, *SHR* and *SCR* was low (Supplementary Figs S6, S7), indicating that these genes are not under the control of DNA demethylation/methylation. In the *CYCB1* promoter, five cytosines showed a reduced frequency of methylation in the *elp2* mutant compared to the WT. The mutant sequence was less methylated at the 3ʹ end of its coding region (Fig. 9). The observed reduced level of methylation in this gene was consistent with its up-regulation in the mutant.

## Discussion

ELP2 is one of a six-subunit (ELP1 to 6) protein complex first isolated from yeast (Wittschieben *et al.*, 1999), and later termed ‘elongator’ on the basis that it was co-purified with the hyper-phosphorylated form of RNA polymerase II. The complex has been shown to support transcription via the regulation of DNA methylation, histone acetylation or tRNA modification (Winkler *et al.*, 2002; Chen *et al.*, 2009; Wang *et al.*, 2013). In plants, its activity has been associated with growth and development, pathogen defence, and responses to abiotic stress. *elp1, elp3 and elp4* have been reported to regulate root growth (Nelissen *et al.*, 2005), although the underlying mechanism of this control has not yet been well elucidated. Here, we demonstrated that *ELP2* also acts as a regulator of root growth, via its effect on the maintenance of the root stem cell niche. The basis of the regulation was the epigenetic modification of the transcription factors *SCR*, *SHR*, *PLT1* and *PLT2*, and the G2/M transition activator *CYCB1*.

### The role of ELP2 in root development

A systematic contrast in root growth and development between WT and the *elp2* loss-of-function mutant showed that the absence of *ELP2* caused root foreshortening as a result of a reduction in cell size in the EZ and in cell number in the MZ. In addition, the mutant was also defective with respect to root stem cell niche maintenance, exhibiting a much increased rate of cell division in the QC and an accelerated differentiation of the root distal stem cells. The mutants *scr*, *shr* and *plt1plt2* were not only compromised with respect to their root growth, but also experienced increased cell division in the QC and accelerated differentiation of their root distal stem cells (Helariutta *et al.*, 2000; Sabatini *et al.*, 2003; Aida *et al.*, 2004; Galinha *et al.*, 2007). A clear hypothesis therefore was that the loss of ELP interfered with the functioning of *SCR*, *SHR*, *PLT1* and *PLT2*. The knockdown of *WOX5*, the product of which is specifically deposited in the QC (Sarkar *et al.*, 2007; Forzani *et al.*, 2014), may also have contributed to the defective root stem cell niche maintenance shown by the *elp2* mutant. It has been established that an optimized level of auxin signalling in the QC is required to ensure root stem cell identity. QC-centered auxin gradients are formed by polar auxin transport and local auxin synthesis (Grieneisen *et al.*, 2007; Tian *et al.*, 2013). Disturbing the optimal level favours accelerated differentiation of the root distal stem cells (Tian et al., 2013, 2014), so a reduction in auxin supply to the mutants’ root tips may also have made a contribution to its root phenotype.

### ELP2 epigenetically affects the transcription of genes involved in root meristem maintenance

The elongator complex acts to acetylate both core histones and nucleosomal substrates (Winkler *et al.*, 2002). In *A. thaliana elp3* mutant, histone H3 lysine 14 acetylation level is reduced in the coding region of both *SHORT HYPOCOTYL 2* (auxin repressor) and *LAX2* (auxin influx carrier), resulting in a reduction in their expression and hence the mutant’s phenotype (Nelissen *et al.*, 2010). The loss of *ELP2* similarly reduces acetylation level in the coding region of the GRAS family transcription factors *SCR* and *SHR*, and that of the AP2 transcription factors *PLT1* and *PLT2*, thereby down-regulating them all. The result underlines the requirement that each of the elongator complex components be present if complex assembly, integrity and enzymatic activity are to proceed normally (Glatt and Muller, 2013). DNA methylation is a central process regulating many of the genes involved in growth and the response to the plant’s environment (Chinnusamy and Zhu, 2009; He *et al.*, 2011; Li *et al.*, 2012; Wang *et al.*, 2013). *ELP2* has been recently shown to contribute to DNA methylation (Wang *et al.*, 2013). Consistent with the generally observed negative correlation between DNA methylation and gene expression, the up-regulation of *CYCB1* in the *elp2* mutant may reflect its reduced level of methylation within both the promoter and/or coding region.

### ELP2 affects auxin signal in root tip

An optimal auxin maximum is required to maintain root stem cell niche identity and root growth (Grieneisen *et al.*, 2007; Tian et al., 2013, 2014). The *elp2* mutant has reduced IAA content in roots, indicating that the ELP2 interferes with the auxin homeostasis regulation. The basal auxin efflux carrier PIN1 drives the flow of auxin from the stele towards the root tip (Galweiler *et al.*, 1998; Blilou *et al.*, 2005; Wisniewska *et al.*, 2006; Grieneisen *et al.*, 2007), while PIN2 controls its flow from the cortex (Blilou *et al.*, 2005; Wisniewska *et al.*, 2006). In the *elp2* mutant, the less polarized PIN1 and decreased levels of both PIN1 and PIN2 reduced the supply of auxin to root tip. Therefore, the *elp2* mutant has reduced auxin signal in the root, which could break the stem cell niche maintenance.

## Supplementary data

Supplementary data are available at *JXB* online.

Supplementary Figure S1. Agarose gel analysis of TAIL-PCR products amplified from WT and *drs1* (*elp2*) genomic DNA

Supplementary Figure S2. The structure of *elp2*.

Supplementary Figure S3. Microgaphs of the root stem cell niche in (A) WT, (B) *elp2-6*, (C), *elp2-7*, (D) *elp1*, (E) *elp4* and (F) *elp6*.

Supplementary Figure S4. *elp* mutants have short primary roots.

Supplementary Figure S5. Histone H3 acetylation levels in (A) *ACTIN2*, (B) *PIN1*.

Supplementary Figure S6. Levels of methylation in (A) *SHR* and (B) *SCR*.

Supplementary Figure S7. DNA methylation level assay of the *PINOID*.

Supplementary Table S1. Summary of primers used in this study.

Supplementary Data

## References

[CIT0001] Aida M Beis D Heidstra R Willemsen V Blilou I Galinha C Nussaume L Noh YS Amasino R Scheres B 2004 The PLETHORA genes mediate patterning of the *Arabidopsis* root stem cell niche. Cell 119, 109–120. 15454085 10.1016/j.cell.2004.09.018

[CIT0002] Benjamins R Quint A Weijers D Hooykaas P Offringa R 2001 The PINOID protein kinase regulates organ development in *Arabidopsis* by enhancing polar auxin transport. Development 128, 4057–4067. 11641228 10.1242/dev.128.20.4057

[CIT0003] Benjamins R Scheres B 2008 Auxin: the looping star in plant development. Annual Review of Plant Biology 59, 443–465. 10.1146/annurev.arplant.58.032806.103805 18444904

[CIT0004] Benkova E Michniewicz M Sauer M Teichmann T Seifertova D Jurgens G Friml J 2003 Local, efflux-dependent auxin gradients as a common module for plant organ formation. Cell 115, 591–602. 14651850 10.1016/s0092-8674(03)00924-3

[CIT0005] Blilou I Xu J Wildwater M Willemsen V Paponov I Friml J Heidstra R Aida M Palme K Scheres B 2005 The PIN auxin efflux facilitator network controls growth and patterning in *Arabidopsis* roots. Nature 433, 39–44. 15635403 10.1038/nature03184

[CIT0006] Chen C Huang B Eliasson M Ryden P Bystrom AS 2011 Elongator complex influences telomeric gene silencing and DNA damage response by its role in wobble uridine tRNA modification. PLoS Genetics 7, e1002258. 21912530 10.1371/journal.pgen.1002258 PMC3164696

[CIT0007] Chen C Tuck S Bystrom AS 2009 Defects in tRNA modification associated with neurological and developmental dysfunctions in *Caenorhabditis elegans* elongator mutants. PLoS Genetics 5, e1000561. 19593383 10.1371/journal.pgen.1000561 PMC2702823

[CIT0008] Chinnusamy V Zhu JK 2009 Epigenetic regulation of stress responses in plants. Current Opinion in Plant Biology 12, 133–139. 19179104 10.1016/j.pbi.2008.12.006 PMC3139470

[CIT0009] Colon-Carmona A You R Haimovitch-Gal T Doerner P 1999 Technical advance: spatio-temporal analysis of mitotic activity with a labile cyclin-GUS fusion protein. The Plant Journal 20, 503–538. 10607302 10.1046/j.1365-313x.1999.00620.x

[CIT0010] Creppe C Malinouskaya L Volvert ML 2009 Elongator controls the migration and differentiation of cortical neurons through acetylation of alpha-tubulin. Cell 136, 551–564. 19185337 10.1016/j.cell.2008.11.043

[CIT0011] Dai M Zhang C Kania U 2012 A PP6-type phosphatase holoenzyme directly regulates PIN phosphorylation and auxin efflux in *Arabidopsis* . The Plant Cell 24, 2497–514. 22715043 10.1105/tpc.112.098905 PMC3406902

[CIT0012] Defraia CT Wang Y Yao J Mou Z 2013 Elongator subunit 3 positively regulates plant immunity through its histone acetyltransferase and radical S-adenosylmethionine domains. BMC Plant Biology 13, 102. 23856002 10.1186/1471-2229-13-102 PMC3728140

[CIT0013] DeFraia CT Zhang X Mou Z 2010 Elongator subunit 2 is an accelerator of immune responses in *Arabidopsis thaliana* . The Plant Journal 64, 511–523. 20807211 10.1111/j.1365-313X.2010.04345.x

[CIT0014] Dello Ioio R Linhares FS Scacchi E Casamitjana-Martinez E Heidstra R Costantino P Sabatini S 2007 Cytokinins determine *Arabidopsis* root-meristem size by controlling cell differentiation. Current Biology 17, 678–682. 17363254 10.1016/j.cub.2007.02.047

[CIT0015] Dello Ioio R Nakamura K Moubayidin L Perilli S Taniguchi M Morita MT Aoyama T Costantino P Sabatini S 2008 A genetic framework for the control of cell division and differentiation in the root meristem. Science 322, 1380–1384. 19039136 10.1126/science.1164147

[CIT0016] Di Laurenzio L Wysocka-Diller J Malamy JE Pysh L Helariutta Y Freshour G Hahn M G Feldmann KA Benfey PN 1996 The SCARECROW gene regulates an asymmetric cell division that is essential for generating the radial organization of the *Arabidopsis* root. Cell 86, 423–433. 8756724 10.1016/s0092-8674(00)80115-4

[CIT0017] Ding Z Friml J 2010 Auxin regulates distal stem cell differentiation in *Arabidopsis* roots. Proceedings of the National Academy of Sciences, USA 107, 12046–12051. 10.1073/pnas.1000672107 PMC2900669 20543136

[CIT0018] Dolan L Janmaat K Willemsen V Linstead P Poethig S Roberts K Scheres B 1993 Cellular organisation of the *Arabidopsis thaliana* root. Development 119, 71–84. 8275865 10.1242/dev.119.1.71

[CIT0019] Forzani C Aichinger E Sornay E Willemsen V Laux T Dewitte W Murray JA 2014 WOX5 suppresses CYCLIN D activity to establish quiescence at the center of the root stem cell niche. Current Biology 24, 1939–1944. 25127220 10.1016/j.cub.2014.07.019 PMC4148176

[CIT0020] Friml J Vieten A Sauer M Weijers D Schwarz H Hamann T Offringa R Jurgens G 2003 Efflux-dependent auxin gradients establish the apical-basal axis of *Arabidopsis* . Nature 426, 147–153. 14614497 10.1038/nature02085

[CIT0021] Galinha C Hofhuis H Luijten M Willemsen V Blilou I Heidstra R Scheres B 2007 PLETHORA proteins as dose-dependent master regulators of *Arabidopsis* root development. Nature 449, 1053–1057. 17960244 10.1038/nature06206

[CIT0022] Galweiler L Guan C Muller A Wisman E Mendgen K Yephremov A Palme K 1998 Regulation of polar auxin transport by AtPIN1 in *Arabidopsis* vascular tissue. Science 282, 2226–2230. 9856939 10.1126/science.282.5397.2226

[CIT0023] Glatt S Muller CW 2013 Structural insights into elongator function. Current Opinion in Structural Biology 23, 235–242. 23510783 10.1016/j.sbi.2013.02.009

[CIT0024] Grieneisen VA Xu J Maree AF Hogeweg P Scheres B 2007 Auxin transport is sufficient to generate a maximum and gradient guiding root growth. Nature 449, 1008–1013. 17960234 10.1038/nature06215

[CIT0025] He XJ Chen T Zhu JK 2011 Regulation and function of DNA methylation in plants and animals. Cell Research 21, 442–465. 21321601 10.1038/cr.2011.23 PMC3152208

[CIT0026] Helariutta Y Fukaki H Wysocka-Diller J Nakajima K Jung J Sena G Hauser MT Benfey PN 2000 The SHORT-ROOT gene controls radial patterning of the *Arabidopsis* root through radial signaling. Cell 101, 555–567. 10850497 10.1016/s0092-8674(00)80865-x

[CIT0027] Huang F Zago MK Abas L van Marion A Galvan-Ampudia CS Offringa R 2010 Phosphorylation of conserved PIN motifs directs *Arabidopsis* PIN1 polarity and auxin transport. The Plant Cell 22, 1129–1142. 20407025 10.1105/tpc.109.072678 PMC2879764

[CIT0028] Kim JH Lane WS Reinberg D 2002 Human elongator facilitates RNA polymerase II transcription through chromatin. Proceedings of the National Academy of Sciences, USA 99, 1241–1246. 10.1073/pnas.251672198 PMC122174 11818576

[CIT0029] Kornet N Scheres B 2009 Members of the GCN5 histone acetyltransferase complex regulate PLETHORA-mediated root stem cell niche maintenance and transit amplifying cell proliferation in *Arabidopsis* . The Plant Cell 21, 1070–1079. 19376933 10.1105/tpc.108.065300 PMC2685635

[CIT0030] Li X Qian W Zhao Y Wang C Shen J Zhu JK Gong Z 2012 Antisilencing role of the RNA-directed DNA methylation pathway and a histone acetyltransferase in *Arabidopsis* . Proceedings of the National Academy of Sciences, USA 109, 11425–11430. 10.1073/pnas.1208557109 PMC3396497 22733760

[CIT0031] Michniewicz M Zago MK Abas L 2007 Antagonistic regulation of PIN phosphorylation by PP2A and PINOID directs auxin flux. Cell 130, 1044–1056. 17889649 10.1016/j.cell.2007.07.033

[CIT0032] Mosher RA Durrant WE Wang D Song J Dong X 2006 A comprehensive structure-function analysis of *Arabidopsis* SNI1 defines essential regions and transcriptional repressor activity. The Plant Cell 18, 1750–1765. 16766691 10.1105/tpc.105.039677 PMC1488919

[CIT0033] Nakajima K Sena G Nawy T Benfey PN 2001 Intercellular movement of the putative transcription factor SHR in root patterning. Nature 413, 307–311. 11565032 10.1038/35095061

[CIT0034] Nelissen H De Groeve S Fleury D 2010 Plant Elongator regulates auxin–related genes during RNA polymerase II transcription elongation. Proceedings of the National Academy of Sciences, USA 107, 1678–1683. 10.1073/pnas.0913559107 PMC2824411 20080602

[CIT0035] Nelissen H Fleury D Bruno L Robles P De Veylder L Traas J Micol JL Van Montagu M Inze D Van Lijsebettens M 2005 The elongata mutants identify a functional elongator complex in plants with a role in cell proliferation during organ growth. Proceedings of the National Academy of Sciences, USA 102, 7754–7759. 10.1073/pnas.0502600102 PMC1140448 15894610

[CIT0036] Petrasek J Mravec J Bouchard R 2006 PIN proteins perform a rate-limiting function in cellular auxin efflux. Science 312, 914–8. 16601150 10.1126/science.1123542

[CIT0037] Rahl PB Chen CZ Collins RN 2005 Elp1p, the yeast homolog of the FD disease syndrome protein, negatively regulates exocytosis independently of transcriptional elongation. Molecular Cell 17, 841–53. 15780940 10.1016/j.molcel.2005.02.018

[CIT0038] Ruzicka K Simaskova M Duclercq J Petrasek J Zazimalova E Simon S Friml J Van Montagu MC Benkova E 2009 Cytokinin regulates root meristem activity via modulation of the polar auxin transport. Proceedings of the National Academy of Sciences, USA 106, 4284–4289. 10.1073/pnas.0900060106 PMC2657394 19246387

[CIT0039] Sabatini S Heidstra R Wildwater M Scheres B 2003 SCARECROW is involved in positioning the stem cell niche in the *Arabidopsis* root meristem. Genes and Development 17, 354–358. 12569126 10.1101/gad.252503 PMC195985

[CIT0040] Sarkar AK Luijten M Miyashima S Lenhard M Hashimoto T Nakajima K Scheres B Heidstra R Laux T 2007 Conserved factors regulate signalling in *Arabidopsis* thaliana shoot and root stem cell organizers. Nature 446, 811–814. 17429400 10.1038/nature05703

[CIT0041] Tian H Niu T Yu Q Quan T Ding Z 2013 Auxin gradient is crucial for the maintenance of root distal stem cell identity in *Arabidopsis* . Plant signaling & Behavior 8, e26429. 24056047 10.4161/psb.26429 PMC4106507

[CIT0042] Tian H Wabnik K Niu T 2014 WOX5-IAA17 feedback circuit-mediated cellular auxin response is crucial for the patterning of root stem cell niches in *Arabidopsis* . Molecular Plant 7, 277–289. 23939433 10.1093/mp/sst118

[CIT0043] Ulmasov T Murfett J Hagen G Guilfoyle TJ 1997 Aux/IAA proteins repress expression of reporter genes containing natural and highly active synthetic auxin response elements. The Plant Cell 9, 1963–1971. 9401121 10.1105/tpc.9.11.1963 PMC157050

[CIT0044] Vanneste S Friml J 2009 Auxin: a trigger for change in plant development. Cell 136, 1005–1016. 19303845 10.1016/j.cell.2009.03.001

[CIT0045] Vanstraelen M Baloban M Da Ines O Cultrone A Lammens T Boudolf V Brown SC De Veylder L Mergaert P Kondorosi E 2009 APC/C-CCS52A complexes control meristem maintenance in the *Arabidopsis* root. Proceedings of the National Academy of Sciences, USA 106, 11806–11811. 10.1073/pnas.0901193106 PMC2710644 19553203

[CIT0046] Vernoux T Benfey PN 2005 Signals that regulate stem cell activity during plant development. Current Opinion in Genetics & Development 15, 388–394. 15967658 10.1016/j.gde.2005.06.008

[CIT0047] Versees W De Groeve S Van Lijsebettens M 2010 Elongator, a conserved multitasking complex? Molecular Microbiology 76, 1065–1069. 20398217 10.1111/j.1365-2958.2010.07162.x

[CIT0048] Wang Y An C Zhang X Yao J Zhang Y Sun Y Yu F Amador D M Mou Z 2013 The *Arabidopsis* elongator complex subunit2 epigenetically regulates plant immune responses. The Plant Cell 25, 762–776. 23435660 10.1105/tpc.113.109116 PMC3608791

[CIT0049] Weingartner M Criqui MC Meszaros T Binarova P Schmit AC Helfer A Derevier A Erhardt M Bogre L Genschik P 2004 Expression of a nondegradable cyclin B1 affects plant development and leads to endomitosis by inhibiting the formation of a phragmoplast. The Plant Cell 16, 643–657. 15004270 10.1105/tpc.020057 PMC385278

[CIT0050] Winkler GS Kristjuhan A Erdjument-Bromage H Tempst P Svejstrup JQ 2002 Elongator is a histone H3 and H4 acetyltransferase important for normal histone acetylation levels in vivo. Proceedings of the National Academy of Sciences, USA 99, 3517–22. 10.1073/pnas.022042899 PMC122555 11904415

[CIT0051] Wisniewska J Xu J Seifertova D Brewer PB Ruzicka K Blilou I Rouquie D Benkova E Scheres B Friml J 2006 Polar PIN localization directs auxin flow in plants. Science 312, 883. 16601151 10.1126/science.1121356

[CIT0052] Wittschieben BO Otero G de Bizemont T Fellows J Erdjument-Bromage H Ohba R Li Y Allis CD Tempst P Svejstrup JQ 1999 A novel histone acetyltransferase is an integral subunit of elongating RNA polymerase II holoenzyme. Molecular Cell 4, 123–128. 10445034 10.1016/s1097-2765(00)80194-x

[CIT0053] Xu D Huang W Li Y Wang H Huang H Cui X 2011 Elongator complex is critical for cell cycle progression and leaf patterning in *Arabidopsis* . The Plant Journal 69, 792–808. 22026817 10.1111/j.1365-313X.2011.04831.x

[CIT0054] Zhou X Hua D Chen Z Zhou Z Gong Z 2009 Elongator mediates ABA responses, oxidative stress resistance and anthocyanin biosynthesis in *Arabidopsis* . The Plant Journal 60, 79–90. 19500300 10.1111/j.1365-313X.2009.03931.x

