# Supplementary material for: The Arabidopsis thaliana elongator complex subunit 2 epigenetically affects root development
Source: J Exp Bot. 2015 May 21;66(15):4631–42. doi: 10.1093/jxb/erv230 (PMC4507768; doi:10.1093/jxb/erv230)
Supplement: Supplementary Data [file supp_66_15_4631__index.html]

The Arabidopsis thaliana elongator complex subunit 2 epigenetically affects root development — Supplementary Data 

# The *Arabidopsis thaliana* elongator complex subunit 2 epigenetically affects root development

## Supplementary Data

Data files

- Supplementary Data - Supplementary Data
- Supplementary Data - Supplementary Data
