# Supplementary material for: The Arabidopsis thaliana elongator complex subunit 2 epigenetically affects root development
Source: J Exp Bot. 2015 May 21;66(15):4631–42. doi: 10.1093/jxb/erv230 (PMC4507768; doi:10.1093/jxb/erv230)
Supplement: Supplementary Data [file supp_erv230_erv230_SuppFigs_SH.pdf]

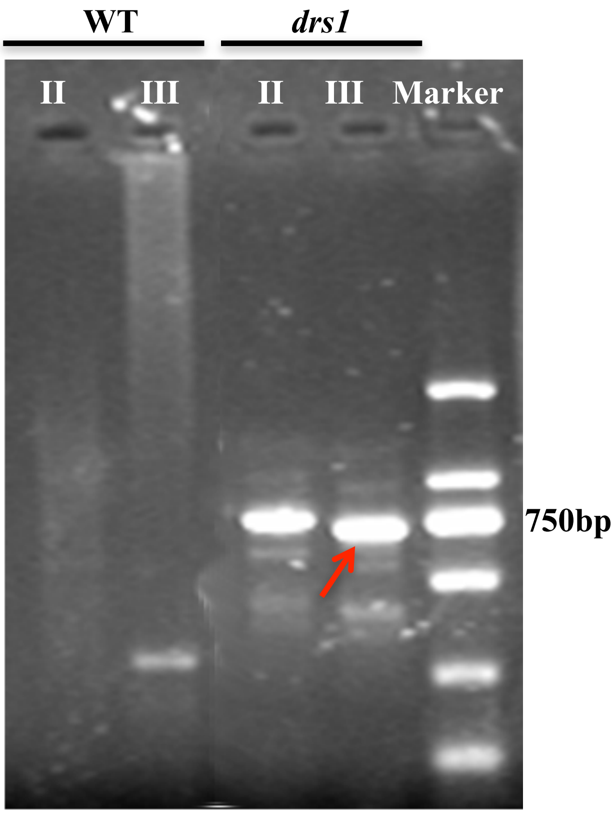

Supplementary Fig. S1. Agarose gel analysis of TAIL-PCR products amplified from WT and *drs1* (*elp2*) genomic DNA. Lanes designated II and III indicate products of, respectively, the secondary and tertiary reactions.

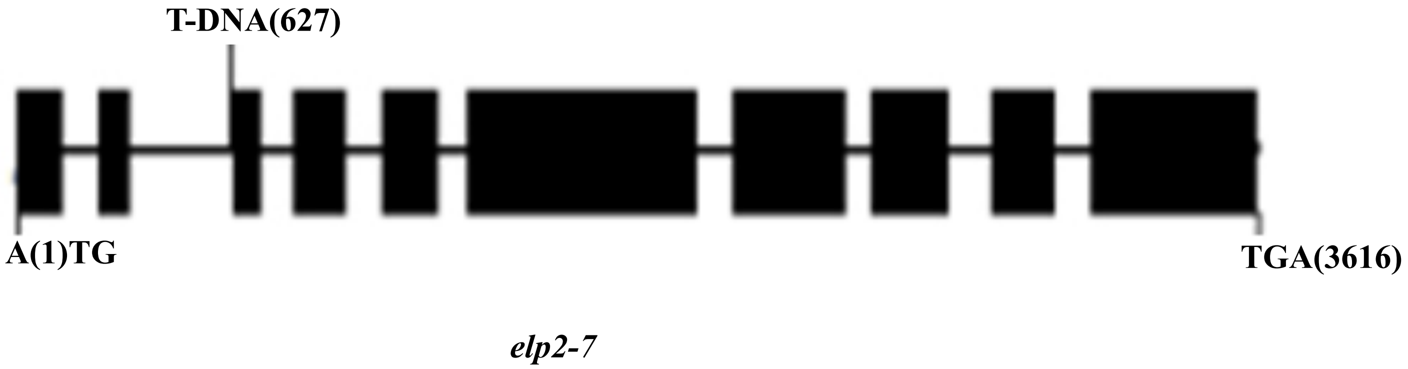

Supplementary Fig. S2. The structure of *elp2*. Boxes and lines represent exons and introns respectively. The position of the T-DNA is indicated.

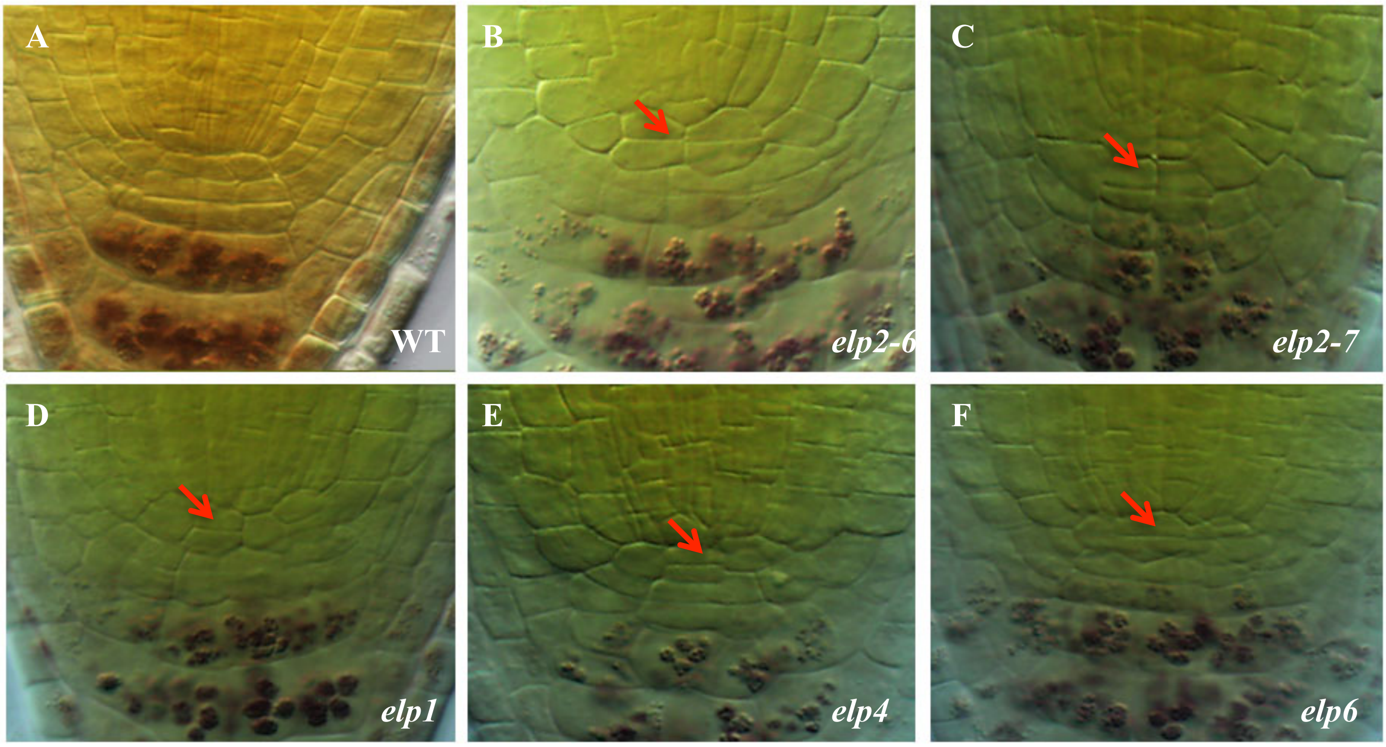

Supplementary Fig. S3. Micrographs of the root stem cell niche in (A) WT, (B) *elp2-6,* (C) *elp2-7,* (D) *elp1*, (E) *elp4* and (F) *elp6*. Cell divisions in the QC indicated by red arrowheads.

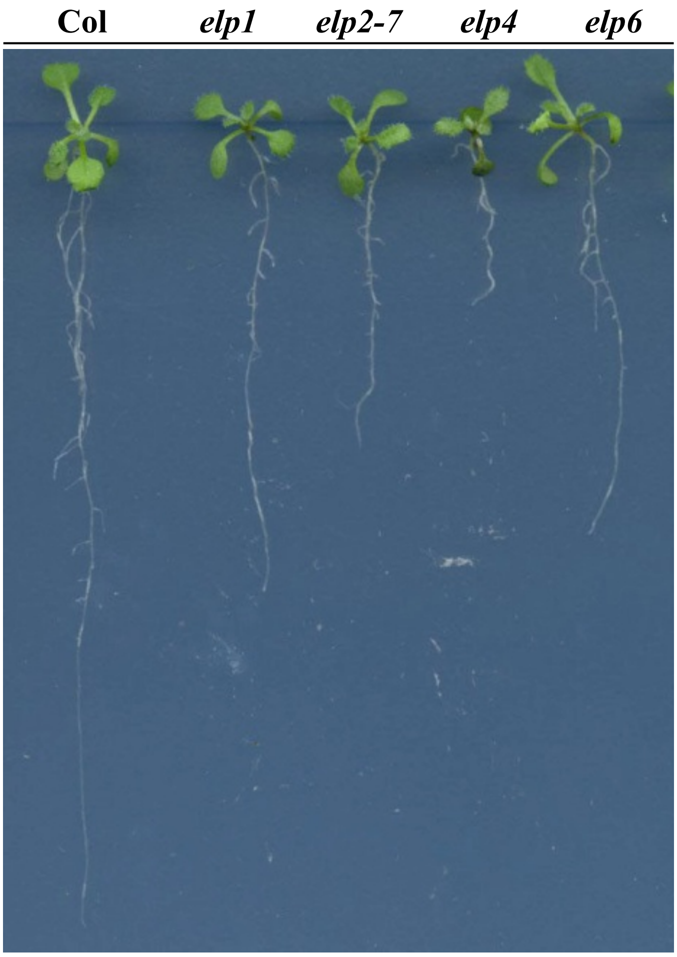

Supplementary Fig. S4. *elp* mutants have short primary roots. From left to right: WT, *elp1*, *elp2-7*, *elp4* and *elp6* seedlings.

Supplement Figure 5

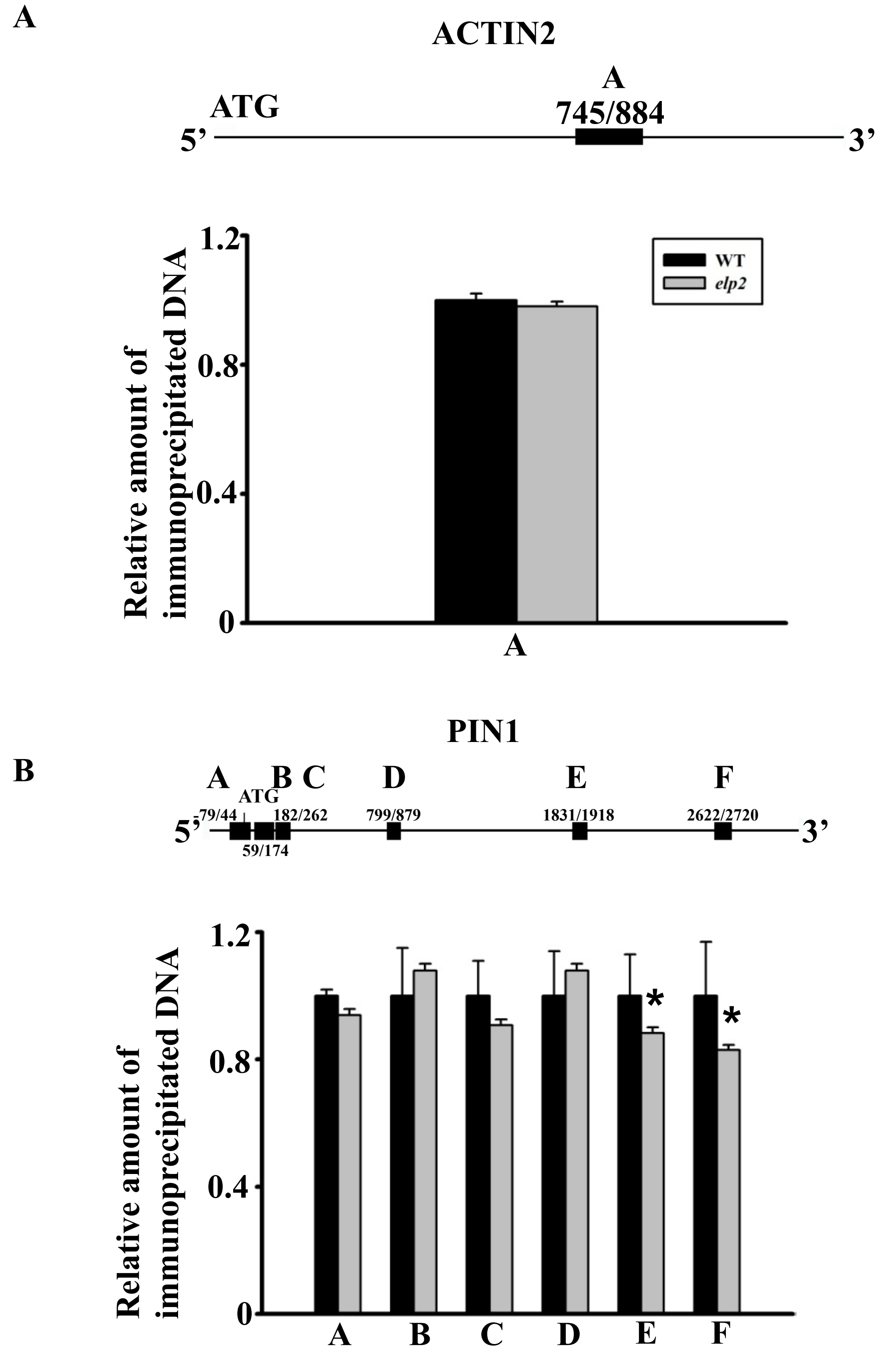

Supplementary Fig. S5. Histone H3 acetylation levels in (A) *ACTIN2*, (B) *PIN1*. The placement of the primers is indicated. The relative amount of immunoprecipitated chromatin fragments in the *elp2* mutant, as determined by qRT-PCR, was compared to that produced in the WT. The data represent mean values of three independent biological repeats with their associated SD (n=3); *, P<0.05.

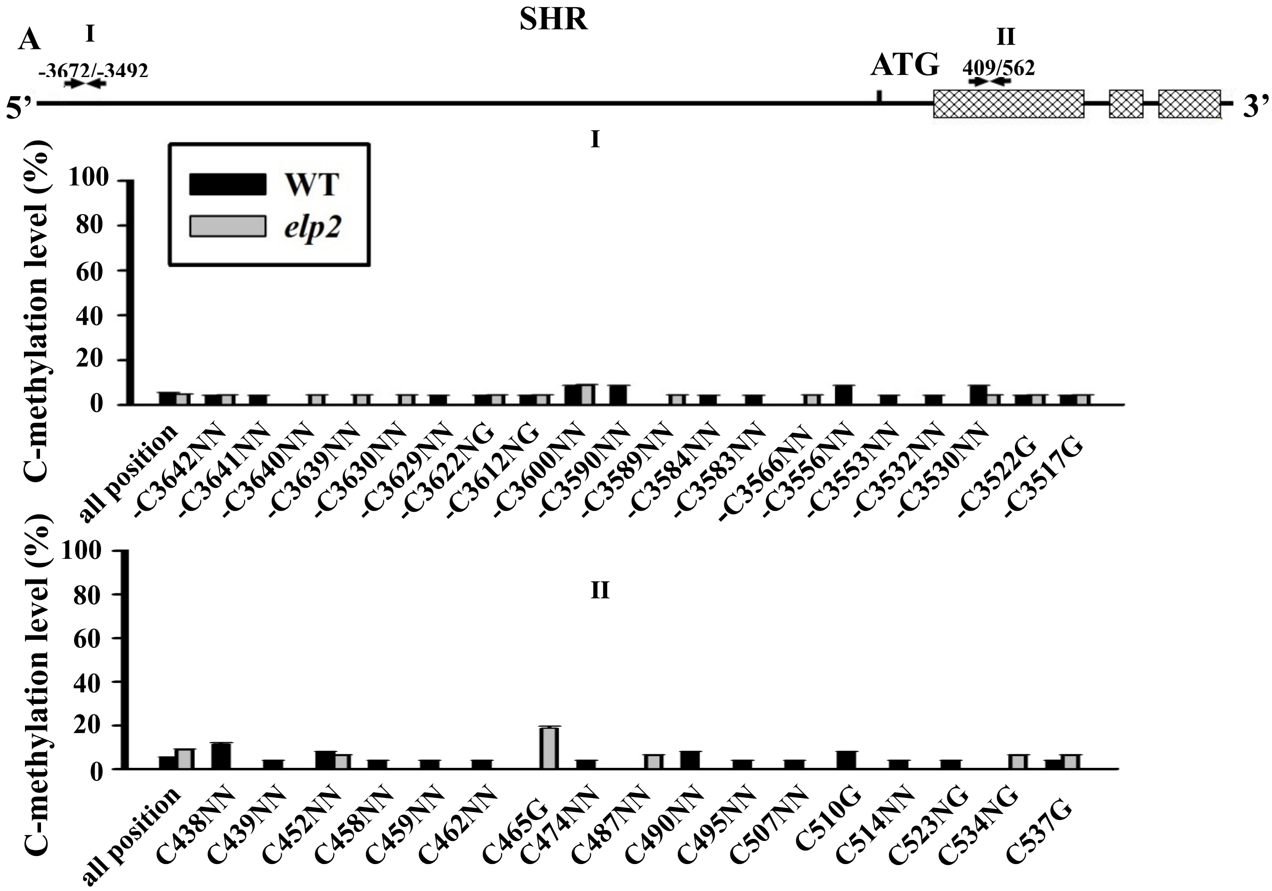

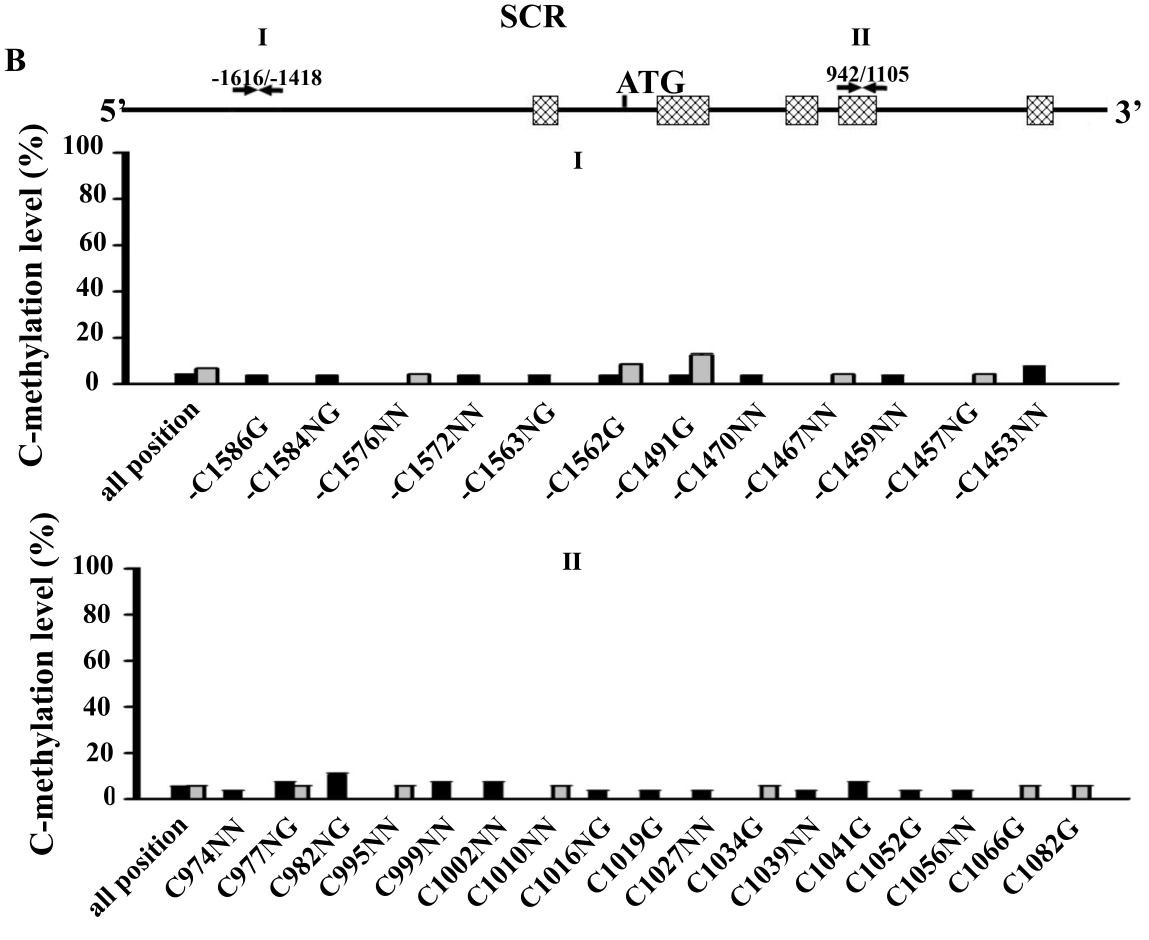

Supplementary Fig. S6. Levels of methylation in (A) *SHR* and (B) *SCR*. 'I' and 'II' represent the position of the primers. GC island in each sequence is shown boxed.

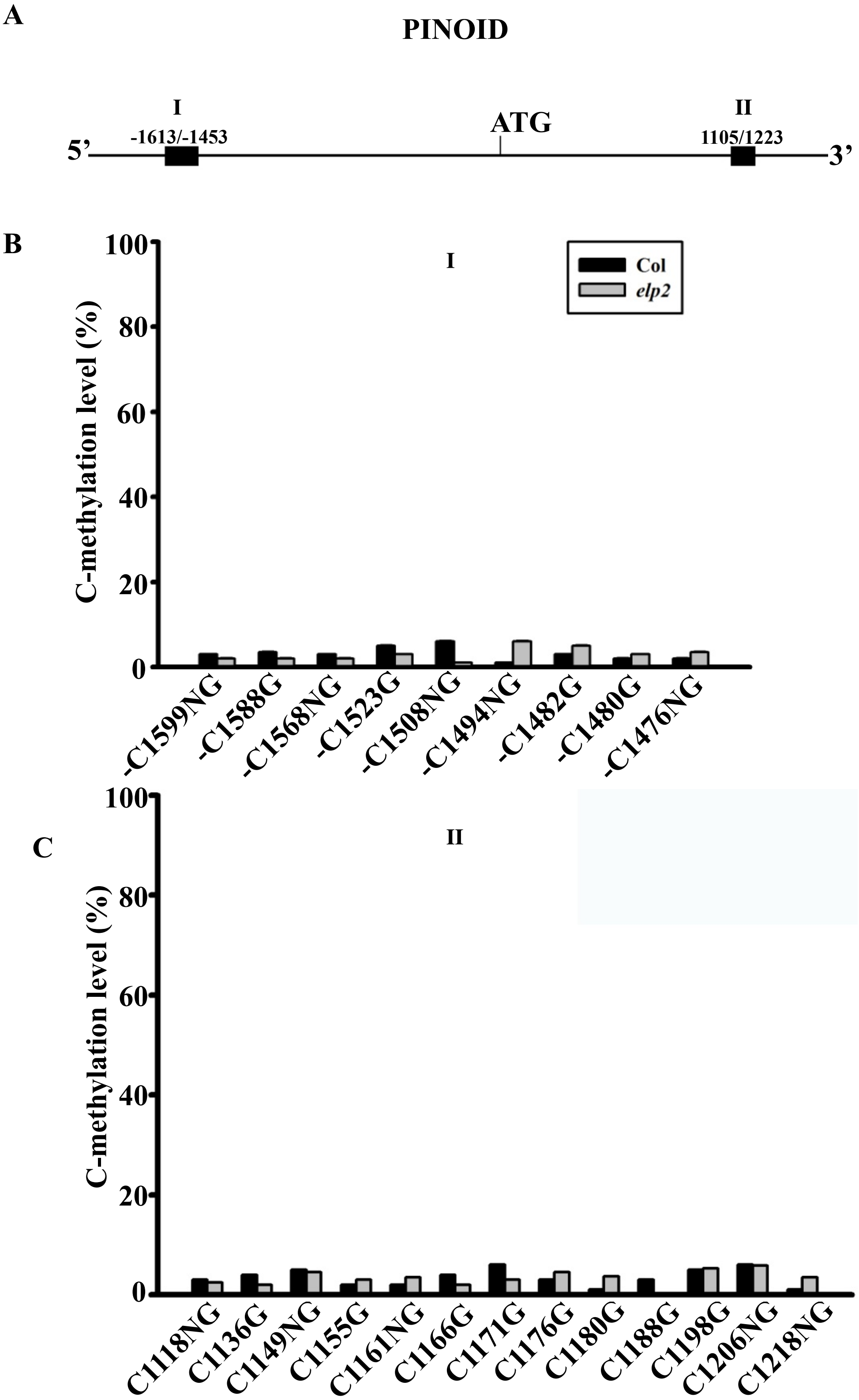

Supplementary Fig. S7. DNA methylation level assay of the *PINOID*. (A) Placement of the primers is indicated. The *PINOID* gene have no CG island. (B, C) The methylation level of amplified fragments I (B), II (C). The DNA was extracted from three biological replicates of both WT and *elp2*. Three replicates of 60 clones derived from each of WT and *elp2* were bisulfite-sequenced to assess their methylation level. The data represent mean values with their associated SD (n=3); *, P<0.05.
