# Supplementary material for: The Arabidopsis thaliana elongator complex subunit 2 epigenetically affects root development
Source: J Exp Bot. 2015 May 21;66(15):4631–42. doi: 10.1093/jxb/erv230 (PMC4507768; doi:10.1093/jxb/erv230)
Supplement: Supplementary Data [file supp_erv230_erv230_SuppTableS1_SH.pdf]

Supplementary Table S1. Summary of primers used in this study

**qRT-PCR primer**	
SHR-Forward	TGGTCGAGGAGGATGAGGAATAG
SHR-Reverse	ACACTGTACCATCGACCAAACACC
SCR-Forward	TAGCGGTTGGAGGACCATCG
SCR-Reverse	CGCTTGTGTAGCTGCATTTCC
CYCB1;1-Forward	CCGGAACTGAATCTGCTTAGG
CYCB1;1-Reverse	GCGACTCATTAGACTTGTTCA
ACT2-Forward	TTGACTACGAGCAGGAGATGG
ACT2-Reverse	ACAAACGAGGGCTGGAACAAG
35SPID-QRT-S	ACACTCTCTCCGTCATAGACAACCT
35SPID-QRT-AS	TAATGTGACCGTCGGATCTAACTAAG
**Acetylation primer**	
PLT1(-69/49)-F	ACCAAAGTGGTAGTGATTTATTGATT
PLT1(-69/49)-R	AAGAAGAGTTGTTCGGTGAAAGAGGA
PLT1(243/363)-F	AGGTTCCAAAAGTGGCCGATTTTCTC
PLT1(243/363)-R	ACGCTAGGCATCAAGCTATTGGTATG
PLT1(1752/1851)-F	CAGAACCAGCCTGTATAAGTGTACG
PLT1(1752/1851)-R	AGCTTCTGCTGCTTCTTCCTCAGT
PLT1(2237/2317)-F	CACAACTTCATCTCCACCAACAGAC
PLT1(2237/2317)-R	ACGCATTGTAGAGCTGCTGAGAGTT
PLT1(2731/2816)-F	GGTAGAAGAATTTAGGTGACGCAAGG
PLT1(2731/2816)-R	AACCCTCAAGAACCATTTAACCAGTC
PLT2(-223/-121)- F	CAGCCATACTTGGAGAAAGCAGAAC
PLT2(-223/-121)- R	TTTGAGAAACGCAAGTTTGGTAAAG
PLT2(444/537) –F	AAAAGTGGCTGATTTCTTAGGAGTG
PLT2(444/537) –R	GGCGTTGGTTTGATGAATGTCGTTATA
PLT2(681/797) –F	TGCAGAAGTCGCCACTGTGAAAGCC
PLT2(681/797) –R	TCCAAAGTCCGTCTCGGTGTAGCCT
PLT2(1273/1367)-F	ACTCAGGTGGGTATGACAAAGAAG
PLT2(1273/1367)-R	CGGAAAGTTGGTAGTAGTAGAGGG
PLT2(2143/2223)-F	ATGATGTGAAAGCCATCCTGGAGAG
PLT2(2143/2223)-R	GAGCTTGAGCTTCTTTGAGCCGTTT
SCR(-960/-843)-F	TGCCCCATCTTAGTAAGCACATCGTA
SCR(-960/-843)-R	ATATGGAGTCAGCAGGTTGCACCACT
SCR(344/538)-F	ACCGTGGTGGTCGGAATGTTATGATGT
SCR(344/538)-R	GAGCACCGAGATTTGGGTTACAAGGGA
SCR (728/859)-F	AAAATTCTTCTACCGATGCACCACC
SCR (728/859)-R	CTTCTTCGTCTTGCTTCTGCCTCTT
SCR(1106/1235)-F	CGTTTCAGGTCTTTAATGGGATAAGC
SCR(1106/1235)-R	AGTCCCTGCATGATGTCCAAGTCAAT
SCR (1235/1359)-F	TTCAATGGCCTGGTTTATTCCACAT
SCR (1235/1359)-R	GAAATCCGAAAGACGTTTCCCTGTA
SCR (1838/1953)-F	TAGCGGTTGGAGGACCATCG
SCR (1838/1953)-R	CGCTTGTGTAGCTGCATTTCC
SCR (1979/2060)-F	CGGATGGTTACACTTTGGTTGATGATA
SCR (1979/2060)-R	CAAGCTGAAGCAGTGAGTAACGAAAGA
SHR(-1610/-1501)-F	GCAGAGAATGGAGAGAGGTTAGGAG

SHR(-1610/-1501)-R	TCATAGGTGTGAGTGAGTGGGAGC
SHR(-1597/-1508)-F	AGAGGTTAGGAGGCAAAGGCA
SHR(-1597/-1508)-R	TGTGAGTGAGTGGGAGCAGG
SHR(274/383)-F	TACCATCCCGCCACATCATCAACC
SHR(274/383)-R	GGTATGGAGAACGCGGAAGGGTCA
SHR(724/868)-F	GCAAACGGAGCAATCTTGGAAGCAGT
SHR(724/868)-R	TGGTTAGCCTTAGGTGAGGCGTGTCG
SHR(1123/1226)-F	ATATCGAGTTTCCGACGGTTAAGAC
SHR(1123/1226)-R	CTCAAGAACTCATCATCAAAGCCAC
SHR(1573/1698)-F	GCTAGTGCGTGGCGGCCAACGTAAAG
SHR(1573/1698)-R	TCTCAATCTCAAAGCCCATCATCAAC
PIN1(-79/44)-F	TTCCCTCTTCACCACTTCTCTCTCA
PIN1(-79/44)-R	ACCATAGCCGTCATAACGTGGTAGA
PIN1(59/174)-F	CTATGATCCTCGCTTACGGCTCTGT
PIN1(59/174)-R	GAAGTGGAAAGAGAGGAGAGGAACG
PIN1(182/262)-F	CTAACAACCCTTACGCCATGAACCT
PIN1(182/262)-R	GGAAGAGGAGAGAGAGGACAATGACTT
PIN1(799/879)-F	GGTGGTCGGAACTCTAACTTTGGTC
PIN1(799/879)-R	TTCGTAGTTGGAAGGTCTCGGAGTAG
PIN1(1831/1918)-F	TGAAATGCCAGCTCTTATAGCAAAGT
PIN1(1831/1918)-R	AAGGAACTGACCAAGACTGAACATAG
PIN1(2622/2720)-F	GGCTATGAGATTTGTCGTTGGACCT
PIN1(2622/2720)-R	ACTACCTGGATAATGGCAACATGGAG
**Methylation primer**	
SHR(409/542)-F	TTAGTTAATGTTAAGTGGGTAGATT
SHR(409/542)-R	AAAAATAAAAAACCAATTTTTACTC
SHR(-3672/-3492)-F	GTTATTTTGTTATGGGAAGTTAGTT
SHR(-3672/-3492)-R	GGATTGATGTTATTGTTTTTGTTA
SCR(942/1105)-F	TGAGATTTTTTAGTTATTAATTTTTTA
SCR(942/1105)-R	CAAAAACCATTTTCAAACTATAC
SCR(-1616/-1418)-F	TTAAAAGGGTAGAAAAGATTATTTAAATAT
SCR(-1616/-1418)-R	CATAACAATAAACCCAAAAAAAA
CYCB1(-697/-524)-F	AAATAAAATAATAGAGGAGGAGTATTATTA
CYCB1(-697/-524)-R	TTATAAATTTACCCCTACAAAAATC
CYCB1(1066/1274)-F	GATTGGTAGAGTGAATGGAGGTTA
CYCB1(1066/1274)-R	AAACCAACAAACTACAATTCTTTTC
CYCB1(1906/2080)-F	ATGAAAAAGGGATTGTGTAAAGTTG
CYCB1(1906/2080)-R	CCTAACTCCTAAACAAATTCAATTCC
PID-PRO-meth-S	TTATATTTTTTTTAATTGTTGTGATT
PID-PRO-meth-AS	AAATTACTCTAATCCAACCTCTATTC
PID-CDS-meth-1-S	GTTTAAAAAGGTTTAGATTTTAGAATTAAT
PID-CDS-meth-1-AS	CACCTAAAACAACTTCTAATACCAC
**AD primer**	
AD1	NGTCGASWGANAWGAA
AD2	TGWGNAGSANCASAGA
AD3	AGWGNAGWANCAWAGG
AD4	STTGNTASTNCTNTGC
AD5	NTCGASTWTSGWGTT
AD6	WGTGNAGWANCANAGA
